# *Enterococcus faecium* supplementation in sows during gestation and lactation improves the performance of sucking piglets

**DOI:** 10.1002/vms3.215

**Published:** 2019-11-25

**Authors:** Ruixia Lan, Inho Kim

**Affiliations:** ^1^ Department of Animal Science College of Agriculture Guangdong Ocean University Zhanjiang Guangdong P.R. China; ^2^ Department of Animal Resource & Science Dankook University Cheonan, Choongnam South Korea

**Keywords:** DSM 7134, growth performance, nutrient digestibility, swine

## Abstract

The aim of this study was to evaluate the effects of *Enterococcus faecium* DSM 7,134 supplementation on the performance of sows and their litters. A total of 15 primiparous sows (Landrace × Yorkshire) were randomly divided into three treatments with five replicates. Dietary treatments were: CON, basal diet; E1, CON + 0.025% *E.* *faecium*; E2, CON + 0.05% *E.* *faecium*. No significant differences were observed on body weight and feed intake of lactating sows with *E.* *faecium* supplementation, but linearly increased the sow apparent total tract digestibility (ATTD) of dry matter (DM), nitrogen (N) and gross energy (GE; *p < *.05), and decreased piglets pre‐weaning mortality (*p < *.05). Piglets from *E.* *faecium*‐supplemented sows linearly increased weaning weight, average daily gain (ADG) and gain:feed ratio (*p < *.05), as well as linearly decreased diarrhoea score (*p < *.05) in the first weaning week. Piglets from *E.* *faecium*‐supplemented sows linearly increased faecal *Lactobacillus* and *Enterococci* counts (*p < *.05), while linearly decreased faecal *Escherichia coli* counts (*p < *.05) after weaning. In conclusion, dietary supplementation of *E. faecium* improved the ATTD of DM, N and GE in lactating sows, as well as improved body weight, ADG and shifted faecal microbiota in their litters.

## Graphical abstract

Dietary supplementation of *Enterococcus faecium* improved digestibility of DM, N and energy in lactating sows, the body weight, ADG and shifted faecal microbiota in piglets.

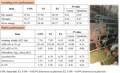

## 1 INTRODUCTION

Probiotics are suggested as desirable antibiotic alternatives to animals by increasing growth performance, nutrient digestibility, enhancing health status and immune regulation (Bontempo, Giancamillo, Savoini, Dell’Orto, & Domeneghini, 2006; Giang, Viet, Ogle, & Lindberg, 2012; Roselli et al., 2005; Stein & Kil, 2006). In swine industry, the most outstanding beneficial effects of probiotics are connected with the competitive exclusion of pathogenic bacteria (Lallès, Bosi, Smidt, & Stokes, 2007). The supplementation of *Enterococcus faecium* to gnotobiotic piglets challenged with *E.* *coli* had fewer diarrhoeas, recovered more quickly and showed anincrease in body weight (Underdahl, 1983). Administration of *E.* *faecium* to weaning pigs had better performance and nutrient utilization (Mallo, Rioperezb, & Honrubiaa, 2010; Zhang, Lee, & Kim, 2014). The potential effects of probiotics alleviate post‐weaning stress has also been studied by supplementing in gestation and lactation sow diets. The neonatal piglet gastrointestinal tract is almost sterile at birth and is colonized by both bacteria acquired from maternal during birth and environmental bacteria (Baker, Davis, Spencer, Moser, & Rehberger, 2013). Previous studies have indicated that neonatal piglets’ gastrointestinal microbiota will shift by supplementation probiotics in sows’ diet (Scharek et al., 2005). Probiotics supplementation in sows’ diet seems to be a useful way in establishing beneficial bacterial species and reducing pathogen load in piglets. However, studies on the effect of *E.* *faecium* supplementation in sows are relatively few.

The body condition of lactation sows is heavily depended on feed intake and nutrient utilization, due to high mobilization during lactation (Nelssen, 1999). Improving feed intake and/or nutrient digestibility may have beneficial effects on sows’ performance. Dietary *E.* *faecium* supplementation had beneficial effects on feed intake and weight performance of primiparous sows (Böhmer, Kramer, & Roth‐Maier, 2006), as well as nutrient digestibility of weaning and growing pigs (Yan & Kim, 2013; Zhang et al., 2014). Lactation sows have severe catabolic conditions, due to massive milk production with limited nutrient intake (Kim and Easter, 2003). If feed intake or/and feed digestibility efficiency are limiting factors leading to catabolic condition, improving nutrient utilization is vital to sow. If the digestibility of nutrient in the conventional sow diet can be improved by dietary *E.* *faecium* supplementation, then total gross energy (GE) available to sows will enhance without increasing feed intake. In addition, the *E.* *faecium* also can transfer to piglets by contact with maternal faeces (Jadamus, Vahjen, & Simon, 2001), the indirect colonization via sow faeces may influence piglet performance and health status (Taras, Vahjen, Macha, & Simon, 2006). Therefore, the objective of this study was to evaluate the effects of *E.* *faecium* supplementation on performance and nutrient digestibility of sows, as well as growth performance and health status of piglets.

## 2 MATERIALS AND METHODS

The experimental protocol used in this study was approved by the Animal Care and Use Committee of Dankook University.

### 2.1 Source of *Enterococcus Faecium*

The *E.* *faecium* DSM 7,134 used in this study was provided by a commercial company (Schaumann Agri International GmbH, Pinneberg, Germany), which is composed of spray‐dried spore‐forming with at least 1.0 × 10^10^ cfu/g of live *E.* *faecium* contained.

### 2.2 Experimental design, animals and housing

A total of 15 sows (Landrace × Yorkshire) were randomly divided into three treatments with five replications. The treatment diets were fed 14 days before farrowing until weaning (weaning at 21 day). Dietary treatments group were: CON, basal diet; E1, CON + 0.025% *E.* *faecium*; E2, CON + 0.05% *E.* *faecium*.

Diets were formulated (Tables 1 and 2) to meet or exceed the nutrient requirements of pigs (NRC, 2012). Sows were feed on a commercial gestation and lactation feed (Table 1) in mash form. During gestation, sows were housed individually in stalls of 2.20 × 0.60 m^2^. The stall had partly slatted floors that consisted of a 0.84 m concrete solid floor and a 1.25 m concrete slatted floor. Approximately 10 day before parturition, sows were moved to farrowing crates, each with 2.20 × 1.80 m^2^. Temperature in the farrowing room was maintained at a minimum of 20°C. Feeds in 1 ml of PBS were serially diluted from 10^–1^ to 10^–7^, and plated on bile esculin azide agar plates in duplicates for 48 hr at 37°C. No *E.* *faecium* counts were detected in the CON diet. The *E.* *faecium* counts were 2.72 × 10^8^ and 2.75 × 10^8^ cfu/kg in the E1 gestation and lactation diet, and 5.40 × 10^8^ and 5.35 × 10^8^ cfu/kg in the E2 gestation and lactation diet.

**Table 1:** Composition of basal sow diets (as‐fed basis)

Items	Gestation diet	Lactation diet
Ingredient, %		
Corn	57.10	51.12
Soybean meal, 46% CP	10.65	24.61
Wheat bran	12.00	4.00
Rice bran	6.00	5.00
Rapeseed meal	3.70	2.50
Tallow	3.59	6.05
Molasses	3.60	3.50
Limestone	0.99	0.76
Dicalcium phosphate	1.52	1.64
Salt	0.60	0.50
L‐Lysine‐HCl, 98%	0.05	0.12
Vitamin premix^a^	0.10	0.10
Mineral premix^b^	0.10	0.10
Calculated composition		
Metabolic energy, MJ/kg	3.19	3.44
Analyzed composition, %		
Crude protein	13.09	17.10
Crude fat	6.88	9.09
Crude fibre	3.21	2.87
Calcium	0.88	0.84
Phosphours	0.76	0.72
Lys	0.65	1.00

a Provided per kilogram of complete diet: vitamin A, 10,000 IU; vitamin D_3_, 2,000 IU; vitamin E, 48 IU; vitamin K_3_, 1.5 mg; riboflavin, 6 mg; niacin, 40 mg; d‐pantothenic, 17 mg; biotin, 0.2 mg; folic acid, 2 mg; choline, 166 mg; vitamin B_6_, 2 mg and vitamin B_12_, 28 μg.

b Provided per kilogram of complete diet: Fe (as FeSO_4_.7H_2_O), 90 mg; Cu (as CuSO_4_.5H_2_O), 15 mg; Zn (as ZnSO_4_), 50 mg; Mn (as MnO_2_), 54 mg; I (as KI), 0.99 mg and Se (as Na_2_SeO_3_.5H_2_O), 0.25 mg.

**Table 2:** Composition of basal weanling pig diet (as‐fed basis)

Items	
Ingredient, %	
Extruded corn	47.39
Soybean meal (Dehulled)	16.00
Fish meal	8.00
Soy oil	2.82
Limestone	0.88
Monocalcium phosphate	0.93
Sweet whey protein	11.10
Lactose	7.60
Plasma powder	4.00
L‐Lysine‐HCl	0.26
DL‐Met	0.27
Threonine	0.15
Choline Chl 50%	0.20
Vitamin premix^a^	0.20
Mineral premix^b^	0.20
Calculated composition	
Metabolic energy, MJ/kg	14.50
Analyzed composition, %	
Crude protein	20.48
Calcium	0.82
Phosphours	0.76
Lys	1.64
Met	0.69

a Provided per kg of complete diet, Vitamin A, 11,025 IU; Vitamin D_3_, 1,103 IU; Vitamin E, 44 IU; Vitamin K, 4.4 mg; Riboflavin, 8.3 mg; Niacin, 50 mg; Thiamine, 4 mg; D‐pantothenic, 29 mg; Choline, 166 mg and Vitamin B_12_, 33 μg.

b Provided per kg of complete diet, Fe (as FeSO_4_.7H_2_O), 80 mg; Cu (as CuSO_4_.5H_2_O), 12 mg; Zn (as ZnSO_4_), 85 mg; Mn (as MnO_2_), 8 mg; I (as KI), 0.28 mg and Se (as Na_2_SeO_3_.5H_2_O), 0.15 mg.

### 2.3 Chemical analysis, sampling and measurements

Gross energy was determined by measuring the heat of combustion in the samples using a bomb calorimeter (Parr 6100; Parr instrument Co.). Dietary dry matter (method 930.15), crude protein (method 968.06), calcium (method 984.01), phosphorus (method 965.17) were analysed according to the procedures described by AOAC International (2005). Individual amino acid composition was measured using an Amino Acid Analyzer (Beckman 6300, Beckman Coulter Inc., Fullerton, CA) after 24‐hr of 6 N‐HCl hydrolysis at 110°C (AOAC International, 2005).

Body weight (BW) and backfat thickness of sows were measured immediately after farrowing and on weaning day. Feed intake was recorded daily to calculate the average daily feed intake (ADFI). The backfat thickness of sows (6 cm off the middle ant the 10th rib) was measured using a real‐time ultrasound instrument (Piglog 105, SFK Technology, Herlev, Denmark). Numbers of born alive or dead were recorded, as well as BW of piglets on day 1, 21, and 35 to calculate average daily gain (ADG) and gain:feed ratio (G:F). Cross‐fostering was performed within 1 day of parturition and among sows of the same treatment. Each litter was standardized to 11 piglets per sow. Creep feed was not given to piglets during the lactation period, and sow milk was the only feed available during lactation. From day 21 to 35, faecal score of weaning pigs was recorded three times per day by the same person, according to the method described by Huang et al. (2015), the scores were as follows: 1 = well‐formed faeces (hard or soft, formed, and moist stool that retains its shape), 2 = sloppy faeces (unformed stool that assumes the shape of the container) and 3 = diarrhoea (liquid stool that can be poured).

To determine the apparent total tract digestibility (ATTD) of dry matter (DM), nitrogen (N) and gross energy (GE), chromium oxide was added to the diets at 2 g/kg, as an indigestible marker (Fenton & Fenton, 1979). Sows were fed the diets for 7‐day, followed by faecal grab sampling via rectal massage. All feed and faecal samples were stored at −20°C until analysis. Before chemical analysis, faecal samples were thawed and dried at 70°C for 72 hr, after which they were finely ground to a size that could pass through a 1‐mm screen. Chromium was analysed by UV absorption spectrophotometry (UV‐1201; Shimadzu, Tokyo, Japan) following the method described by Williams, David, and Iismaa (1962). The digestibility was calculated according to the following formula: ATTD = [1 − {(N_f_ × C_d_)/ (N_d_ × C_f_)}], Where N_f_ = nutrient concentration in faeces (%DM), N_d_ = nutrient concentration in diets (%DM), C_f_ = chrome concentration in faeces (%DM) and C_d_ = chrome concentration in diets (%DM). Gross energy was determined by measuring the heat of combustion in the samples using a bomb calorimeter (Parr 6100; Parr instrument Co.).

For microbiota analysis, at weaning day, faecal samples were collected from five sows and five piglets (one piglet per sow) from each treatment. At day 14 of weaning, faecal samples were collected from five weaning pigs (one weaning pig per sow) from each treatment. The faecal samples were placed on ice and transportation to the laboratory where analysis was immediately carried out according to the method described by Böhmer et al. (2006). One gram of faecal samples was diluted with 9 ml of 1% peptone broth to the dilution step from 10^–1^ to 10^–7^. The specimens were tested for faecal *Lactobacilli*, *E.* *coli* and *E*nterococci counts. The bacterial counts were performed by the spread‐plate procedure on three different culture media (*Lactobacilli* medium III agar, MacConkey agar and Slanetz‐Bartley agar, respectively). *Lactobacilli* were incubated for 72 hr at 37°C in an oxygen‐free atmosphere. *E.* *coli* and *Enterococci* were incubated for 48 hr at 37°C in an oxygen atmosphere. The microbial populations were counted after removing from the incubator, and log transformed before statistical analysis.

Blood samples were collected via jugular venipuncture into clot activator vacuum tubes (Becton Dickinson Vacutainer Systems) from sows and five piglets (one piglet per sow) at weaning day. Lymphocyte was analysed by automatic blood analyser (ADVIA 120, Bayer). IgA, IgG and IgM concentration were analysed using commercial kits purchased from Nanjing Jiancheng Institute of Bioengineering.

### 2.4 Statistical analysis

Both sow and piglet performance data were analysed with SAS 2003 (v. 9.1, SAS Institute Inc.) using the mixed GLM procedure. Sow BW and backfat data were analysed using a repeated measurement method. The method included diet as a fixed effect whereas sow and period were included as random effects. The sows were used as the experiment unit. Piglets birthweight was used as covariates for weaning weights during lactation. Lactation length was used as a covariate for number of piglet survivability, sows and piglets weaning weight, sow BW loss, ADFI and backfat thickness loss, and piglets and weaning pig ADG. Before conducting statistical analysis of the faecal microbiota counts, a logarithmic conversion of the data was performed. Orthogonal comparison was examined using polynomial regression to measure the linear and quadratic effects of increasing concentration of *E.* *faecium*. Statistically significant difference was satisfied when *p* < .05.

## 3 RESULTS

### 3.1 Growth performance and nutrient digestibility in lactating sows

Dietary *E.* *faecium* supplementation had no significant differences in BW, BW loss, feed intake or backfat thickness loss of sows (Table 3). Linear and quadratic effects were observed in pre‐weaning mortality (*p < *.05) of piglets with the increasing level of *E.* *faecium* supplementation, and linear effects were observed in ATTD of dry matter, nitrogen and energy (*p < *.05; Table 4).

**Table 3:** Effects of *Enterococcus faecium* supplementation on performance in sows

Item	CON	E1	E2	*SE*	*p‐value*	
					Linear	Quadratic
Sows						
Live weight, kg						
After farrowing	252.18	256.00	250.86	6.94	0.89	0.60
Weaning	241.26	246.74	240.62	7.07	0.95	0.51
Live weight loss during lactation	10.92	9.26	10.24	0.61	0.37	0.08
Average daily feed intake, kg/d						
Gestation	2.47	2.47	2.47	‐	‐	‐
Lactation	6.10	6.20	6.21	0.21	0.75	0.68
Backfat thickness, mm						
After farrowing	20.88	21.70	21.50	0.33	0.14	0.16
Weaning	18.50	19.50	19.10	0.31	0.18	0.09
Backfat thickness loss	2.38	2.20	2.40	0.30	0.93	0.58
Weaning to estrus interval, d	4.26	4.40	4.20	0.11	0.95	0.49
Number of piglets born alive	11.80	11.20	11.00	1.11	0.57	0.84
Number of weaned piglets	10.25	10.40	10.20	0.18	0.84	0.43
Pre‐weaning mortality, %	13.14	7.14	7.27	0.32	<0.00	<0.00

CON, basal diet; E1, CON + 0.025% *E.* *faecium*; E2, CON + 0.05% *E.* *faecium*; *SE*, Standard error.

**Table 4:** Effects of *Enterococcus faecium* supplementation on nutrient digestibility in lactating sows

Item	CON	E1	E2	*SE*	*p‐value*	
					Linear	Quadratic
Dry matter	65.01	67.09	68.18	0.69	0.01	0.69
Nitrogen	70.17	72.05	73.56	0.93	0.02	0.87
Gross energy	65.39	66.48	69.17	0.92	0.01	0. 50

CON, basal diet; E1, CON + 0.025% *E.* *faecium*; E2, CON + 0.05% *E.* *faecium*; *SE*, Standard error.

### 3.2 Growth performance and faecal score in piglets

On weaning day, BW and ADG of piglets were higher in sows receiving the *E.* *faecium* supplemented diets compared with the CON diet (Table 5). Similarly, during day 22 to 35, linear trend were observed in ADG (*p < *.10) and linear effects were observed in G:*F* (*p < *.05) with *E.* *faecium* supplementation. During day 22 to 28, diarrhoea score linearly decreased (*p < *.05) with increasing levels of *E.* *faecium* supplementation*.*

**Table 5:** Effects of *Enterococcus faecium* supplementation on performance in piglets

Item	CON	E1	E2	*SE*	*p*‐value	
					Linear	Quadratic
Piglets (day 1 to 21)						
Initial weight, kg	1.47	1.42	1.41	0.04	0.28	0.77
Weaning weight, kg	7.54	7.99	8.19	0.07	<0.00	0.18
ADG, g	233.49	252.60	261.07	2.85	<0.00	0.14
Weaning pigs (day 22 to 35)						
ADG, g	293.57	302.91	311.05	6.45	0.06	0.94
ADFI, g	353.63	351.64	350.32	3.54	0.51	0.94
G:F	0.83	0.86	0.88	0.02	0.04	0.86
Diarrhoea score^a^						
Weaning week 1 (day 22–28)	1.93	1.60	1.47	0.15	0.03	0.59
Weaning week 2 (day 29 to 35)	1.47	1.33	1.20	0.13	0.16	1.00

CON, basal diet; E1, CON + 0.025% *E. faecium*; E2, CON + 0.05% *E.* *faecium*; *SE*, Standard error.

a Diarrhoea scores: 1–3, where 1 = well‐formed faeces (hard or soft, formed, and moist stool that retains its shape), 2 = sloppy faeces (unformed stool that assumes the shape of the container) and 3 = diarrhoea (liquid stool that can be poured).

### 3.3 Faecal microbiota and blood immune parameters in lactating sows and piglets

**Figure 1:**
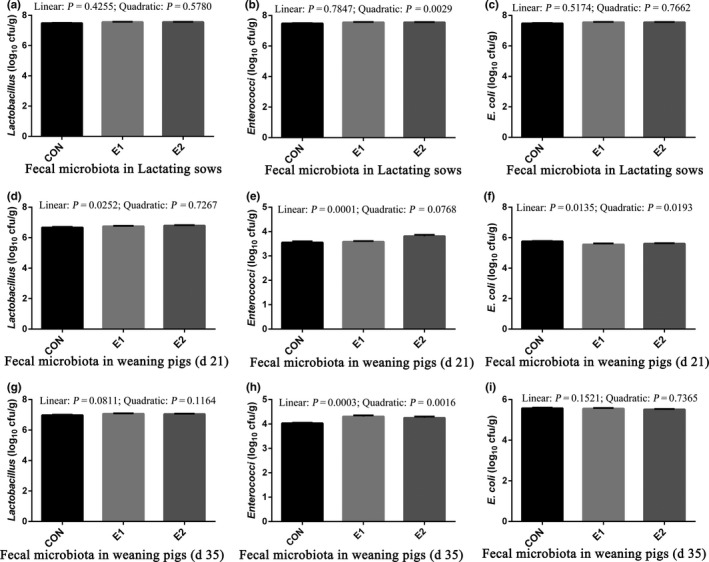
Effects of *Enterococcus faecium* supplementation on microbiota in lactation sows and piglets. Values are mean ± standard error. CON, basal diet; E1, CON + 0.025% *E. faecium*; E2, CON + 0.05% *E. faecium*. (a) *Lactobacillus* in Lactating sows (b) *Enterococci* in Lactating sows (c) *E.coli* in Lactating sows (d) *Lactobacillus* in weaning pigs on day 21 (e) *Enterococci* in weaning pigs on day 21 (f) *E.coli* in weaning pigs on day 21 (g) *Lactobacillus* in weaning pigs on day 35 (h) *Enterococci* in weaning pigs on day 35 (i) *E.coli* in weaning pigs on day 35

In lactating sows, a quadratic effect was observed in *Enterococci* counts (*p < *.05), while no differences were observed in faecal *Lactobacillus* or *E. coli* counts among treatments (Figure 1a–c). On weaning day, *E. faecium* suplementation linearly increased fecal *Lactobacillus* and *Enterococci* counts (*p* < .05) in piglets, but linearly decreased *E.coli* counts (*p* < .05; Figure 1d–f). On day 35, *E. faecium* suplementation linearly increased fecal *Enterococci* counts (*p* < .05) in piglets, and an increasing trend in *Lactobacillus* counts (*p* < .10; Figure 1g–I).

No differences were observed in blood immune parameters of sows and piglets with *E.* *faecium* supplementation (Table 6).

**Table 6:** Effects of *Enterococcus faecium* supplementation to sows on blood immune parameters in sows and piglets

Item	CON	E1	E2	*SE*	*p‐value*	
					Linear	Quadratic
Lactating sows						
IgG, mg/dl	209.60	209.80	206.60	8.55	0.35	0.77
IgA, mg/dl	42.60	43.0 0	42.80	2.48	0.70	0.76
IgM, mg/dl	31.20	32.20	32.80	3.14	0.74	0.96
Lymphocyte, %	44.09	46.53	46.22	4.59	0.69	0.76
Piglets						
IgG, mg/dl	213.20	213.80	238.00	18.54	0.47	0.55
IgA, mg/dl	38.60	43.40	42.80	1.80	0.26	0.34
IgM, mg/dl	30.60	31.80	31.20	1.65	0.78	0.90
Lymphocyte, %	45.65	44.88	45.44	3.49	0.73	0.73

CON, basal diet; E1, CON + 0.025% *E.* *faecium*; E2, CON + 0.05% *E.* *faecium*; *SE*, Standard error.

## 4 DISCUSSION

In this study, the BW, BW loss, ADFI and backfat thickness of sows were not influenced by dietary *E.* *faecium* supplementation, however, decreased pre‐weaning mortality. For piglets, optimizing the gastrointestinal ecosystem and nutrient management seems of utmost importance to maintain piglet performance and health status (Taras et al., 2006). Probiotics can transfer to piglets by contact with maternal faeces (Jadamus et al., 2001), which may be accompanied by the beneficial effects on faecal microbiota, immunogenic factors and diarrhoea incidence (Schanler, 2000). The increased faecal *Lactobacillus* and *Enterococci* counts, and decreased *E. coli* counts of piglets on weaning day may explain the decreased pre‐weaning mortality with dietary *E.* *faecium* supplementation. During lactation, body loss in sows is mainly due to high milk yield and relatively low feed intake (Lallès et al., 2007), the adequate feed intake for lactation sows is crucial to guarantee their performance. Alexopoulos et al. (2004) reported that there was an increase in feed intake and a decrease in weight loss in lactating sow with *B. licheniformis* and *B. subtilis* blend supplementation. Other studies also confirmed the decreased weight loss of sow with probiotics supplementation during lactation (Kreuzer & Zerhusen, 1995). However, no significant differences were observed in weight loss or feed intake with *E.* *faecium* supplementation in this study. The different results may be due to different probiotics strain used, dose level and diet composition.

In this study, BW and ADG of weaning pigs were linearly increased with the increasing level of *E.* *faecium* in the diet of lactating sow. Similar results were also reported by Alexopoulos et al. (2004), who indicated that sow administrated with *Bacillus* and *E.* *faecium* had lower pre‐weaning mortality and higher weaning weight in piglets. Taras et al. (2006) reported the administration of *E.* *faecium* to sows and their piglets led to decreased piglet mortality and reduced pre‐ and post‐weaning diarrhoea. Baker et al. (2013) also reported that sows with *Bacillus* supplementation improved litter weaning weight, ADG, and decreased mortality. The improved BW and ADG of piglets in this study may be due to improved nutrient digestibility of sows, and finally lead to better milk production. We know that there is a strong relationship between piglets’ BW gain and milk production and constituent (Noblet, Dourmad, & Etienne, 1990). However, milk production and constituent were not measured here, which is the limitation of this study.

Dietary *E.* *faecium* supplementation linearly increased the ATTD of DM, N and GE in lactating sows, which was consistent with Zhang et al. (2014), who reported that the ATTD of N and GE was enhanced in weaning pigs with *E.* *faecium* supplementation. Yan and Kim (2013) also reported that dietary *E.* *faecium* supplementation increased the ATTD of DM, N and GE in growing pig. *E.* *faecium* is a normal microorganism in swine intestine, which produce lactic acid to reduce intestinal pH and inhibit the load of invasive pathogens (Canibe & Jensen, 2003), thus may be a reason to explain the improving nutrient digestibility in this study.

The gastrointestinal and lymphoid systems are the largest immunologically competent organs, the development and composition of the gastrointestinal microbiota are the principal factors influencing maturation and optimal development of immunologically systems (Cho & Kim, 2014). In this study, *E.* *faecium* supplementation showed only minor changes in the gut of sows with a slight reduction in *E. coli* counts and a slight increase in *Lactobacillus* counts. The gastrointestinal flora of adult sows has stabilized, unlike piglet, a fundamental change by using probiotics is unlikely (Gedek, 1993), which was confirmed by Sarabia, Villar, Magboo, and Roxas (1997).

The piglet gut is sterile in utero and becomes colonized after birth mainly by bacteria acquired from the sow and sow faeces (Mackie et al., 1999). The early development of the gastrointestinal microbiota and colonization by environmental bacteria have long‐term effects on the host and immune development of the neonate (Tannock, 2005; Thompson, Wang, & Holmes, 2008), as well as regulates host metabolism, growth and susceptibility to disease (Konstantinov et al., 2006; Marques et al., 2010; Turnbaugh et al., 2006). Previous studies reported the transfer of *Bacillus* from sow to piglet via the faecal‐oral route (Baker, Davis, Spencer, Moser, & Rehberger, 2008). In this study, *E.* *faecium* supplementation to sow diets, a linear decrease was detected in faecal *E. coli* counts in piglets on weaning day. In addition, faecal *Lactobacillus* and *Enterococci* counts were linearly increased in piglets from *E.* *faecium* ‐supplemented sows indicating that the microbial colonization shifted from sows to piglets.

Blood lymphocyte, IgG, IgA and IgM concentration are regularly checked to evaluate the humoral immune status of animals. Former studies on the effect of *E.* *faecium* on the immune response of sows and piglets are not always consistent. No significant differences in intestinal IgA or serum IgG were observed with *E.* *faecium* supplementation (Broom, Miller, Kerr, & Knapp, 2006; Scharek, Guth, Filter, & Schmidt, 2007). In this study, no differences were observed in serum IgG, IgA, IgM or lymphocyte concentration with *E.* *faecium* supplementation. However, Szabó et al. (2009) reported that *E.* *faecium* supplementation to weaning piglets challenged with *Salmonella Typhimurium* had higher serum IgM and IgA concentration, it was not sure whether the increased IgM and IgA concentration was a result of *E.* *faecium* supplementation or a result of elevated *Salmonella* loads. Relatively fewer studies have been done to evaluate the effects of *E.* *faecium* on immune status of sows and piglets, more studies are need do to evaluate the mechanism of *E.* *faecium* on immune response in the future.

## 5 CONCLUSION

Our studies suggested that the supplementation of *E.* *faecium* in the diet of gestation and lactating sows had no significant effects on BW, BW loss, ADFI and backfat thickness of sows, but decreased pre‐weaning mortality, improved BW, ADG and shifted faecal microbiota in piglets, as well as improved the digestibility of DM, N and GE in lactating sow.

## CONFLICT OF INTEREST

All authors have no potential conflict of interest to statement.

## ETHICAL STATEMENT

The authors confirm that the ethical policies of the journal, as noted on the journal's author guidelines page, have been adhered to and the appropriate Ethical Review Committee approval has been received. The Korean National Research Council's guidelines for the Care and Use of Laboratory Animals were followed.
